# Kβ X‐Ray Emission Spectroscopic Study of a Second‐Row Transition Metal (Mo) and Its Application to Nitrogenase‐Related Model Complexes

**DOI:** 10.1002/anie.202003621

**Published:** 2020-05-29

**Authors:** Rebeca G. Castillo, Justin T. Henthorn, Jeremy McGale, Dimitrios Maganas, Serena DeBeer

**Affiliations:** ^1^ Department of Inorganic Spectroscopy Max Planck Institute for Chemical Energy Conversion Stiftstrasse 34–36 45470 Mülheim an der Ruhr Germany; ^2^ Max-Planck-Institut für Kohlenforschung Kaiser-Wilhelm-Platz 1 45470 Mülheim an der Ruhr Germany

**Keywords:** molybdenum, nitrogenase, valence-to-core, X-ray emission spectroscopy

## Abstract

In recent years, X‐ray emission spectroscopy (XES) in the Kβ (3p‐1s) and valence‐to‐core (valence‐1s) regions has been increasingly used to study metal active sites in (bio)inorganic chemistry and catalysis, providing information about the metal spin state, oxidation state and the identity of coordinated ligands. However, to date this technique has been limited almost exclusively to first‐row transition metals. In this work, we present an extension of Kβ XES (in both the 4p‐1s and valence‐to‐1s [or VtC] regions) to the second transition row by performing a detailed experimental and theoretical analysis of the molybdenum emission lines. It is demonstrated in this work that Kβ_2_ lines are dominated by spin state effects, while VtC XES of a 4d transition metal provides access to metal oxidation state and ligand identity. An extension of Mo Kβ XES to nitrogenase‐relevant model complexes shows that the method is sufficiently sensitive to act as a spectator probe for redox events that are localized at the Fe atoms. Mo VtC XES thus has promise for future applications to nitrogenase, as well as a range of other Mo‐containing biological cofactors. Further, the clear assignment of the origins of Mo VtC XES features opens up the possibility of applying this method to a wide range of second‐row transition metals, thus providing chemists with a site‐specific tool for the elucidation of 4d transition metal electronic structure.

## Graphical abstract

**The first systematic** Kβ XES study for a second‐row transition metal, including a thorough analysis of the transitions responsible for Kβ_2_ (4p→1s) and valence‐to‐core (valence→1s) emission lines, is presented. Mo–Fe–S cubane clusters are also explored as a practical example of the efficiency of this technique for elucidating the nature of not only Mo but also any other second‐row TM center in biological systems.

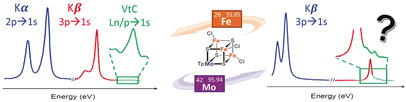

## Introduction

Molybdenum plays crucial roles in biological, geochemical, and catalytic cycles.^1^, ^2^, ^3^, ^4^, ^5^, ^6^, ^7^ Enzymatic systems containing molybdenum are responsible for a wide variety of catalytic functions, including oxygen atom transfer and hydroxylation reactions, as well as the mediation of oxidation/reduction reactions in nitrogen, sulfur, and carbon metabolism. In most of the preceding cases, the catalytically active sites are comprised of mononuclear Mo atoms coordinated by a tricyclic pterin cofactor.^7^, ^8^ To date, only two Mo enzymes have been identified that do not incorporate a pterin cofactor—the Mo‐dependent nitrogenases, which utilize a Mo–7Fe–9S–C cluster to effect nitrogen reduction,^9^, ^10^ and the orange protein, which contains a unique heterometallic Mo–Cu sulfide cluster of unknown function.^11^

Mo K‐edge X‐ray absorption spectroscopy (XAS) and extended X‐ray absorption fine structure (EXAFS) studies have had a significant impact on our understanding of the geometric and electronic structure of the active sites in Mo‐containing enzymes.^12^, ^13^, ^14^, ^15^, ^16^, ^17^, ^18^, ^19^, ^20^, ^21^, ^22^, ^23^, ^24^ However, Mo Kβ X‐ray emission spectroscopy (XES) of enzymatic systems has yet to be explored, and relatively little Kβ XES has been reported for any of the 4d transition metals (TMs).^25^, ^26^, ^27^, ^28^ This is in stark contrast to 3d TMs, where Kβ XES has been utilized for the entire 1^st^ transition series^26^, ^29^, ^30^, ^31^, ^32^, ^33^, ^34^, ^35^, ^36^, ^37^ and applied in a wide range of fields, including homogenous and heterogeneous catalysis, materials science, geochemistry, and bioinorganic chemistry.^32^, ^36^, ^38^, ^39^, ^40^, ^41^, ^42^, ^43^, ^44^, ^45^, ^46^, ^47^, ^48^ Kβ XES of 1^st^ row TMs has seen increasing use as an experimental probe of both metal spin state, based on the Kβ mainlines (3p‐1s) region, and local coordination environment, based on the valence‐to‐core (VtC, ligand to 1s) region.

**Figure 1:**
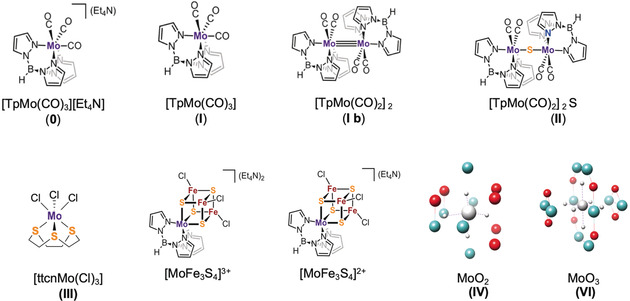
Model compounds investigated in the present study.

However, parallel studies on 4d TM complexes are exceedingly limited. Herein, we perform a detailed Kβ XES study on a series of Mo complexes, shown in Figure 1 and Table 1. While this work is mainly focused on Mo Kβ XES, we also present Mo Kβ_1_ high‐energy resolution fluorescence detected (HERFD) XAS as a complementary electronic structure probe.

**Table 1:** Oxidation states and spin states of model complexes investigated in this study.

Compound (label)	Mo Oxidation state	Mo local spin state
[TpMo(CO)_3_][Et_4_N] (0)	0	0
[TpMo(CO)_3_] (I)	I	1/2
[TpMo(CO)_2_]_2_ (Ib)	I,I	1/2,1/2
[Tp_2_Mo_2_(CO)_4_](μ‐S) (II)	II,II	1,1
[ttcnMo(Cl)_3_] (III)	III	3/2
[MoFe_3_S_3_]^3+^	III	1/2
[MoFe_3_S_3_]^2+^	III	1/2
[MoO_2_] (IV)	IV	1
[MoO_3_] (VI)	VI	0

In order to better understand the significance of the results presented here, it is useful to first briefly summarize the previous Kβ XES work done on 3d TMs and to compare this to the existing assignments for Kβ XES on 4d TMs.

**Figure 2:**
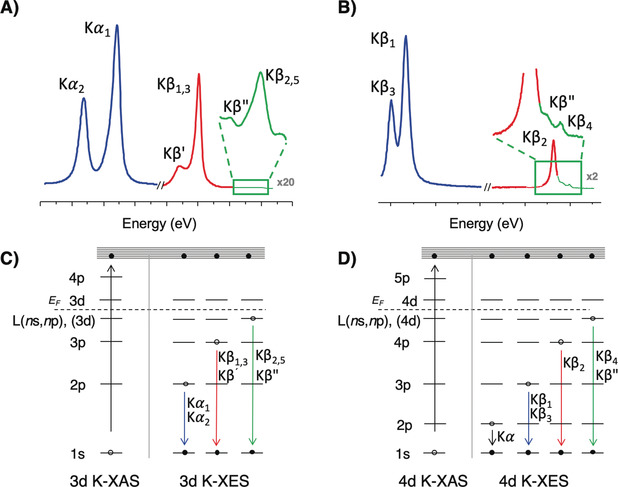
X‐ray emission spectra and energy level diagrams of the absorption and emission processes for 3d (A and C) and 4d (B and D) TMs.

**X‐ray emission spectroscopy, 1^st^ vs. 2^nd^ row transition metals**. Figure 2 shows a comparison between the emission lines of 3d (A) and 4d (B) TMs. Figure 2 A includes the well‐established emission lines in increasing energy order: Kα_2_, Kα_1_, Kβ′, Kβ_1,3_ and the VtC transitions (Kβ′′ and Kβ_2,5_). A representative energy level diagram is displayed below the emission spectrum in Figure 2 C. Figure 2 C shows the non‐resonant emission processes that result from the ionization of the 1s core electron to the continuum. The analogous processes for 4d TMs are shown in Figure 2 B and D. In the present study, we focus on the Kβ_3_, Kβ_1_, Kβ_2_ Kβ′′, and Kβ_4_ emission lines, in increasing energy order.

The Kβ_1_ and Kβ_3_ emission lines in Mo XES correspond to the electric dipole allowed 3p→1s transitions. The splitting of these features is due to 3p core hole spin–orbit coupling (SOC, ≈18 eV splitting). This may be contrasted with the Kβ mainline XES of the 3d TMs, which splits into Kβ_1,3_ and Kβ′ features predominantly due to 3p–3d exchange coupling, with the 3p SOC (<1 eV)) remaining as only a minor perturbation.

To ≈300 eV higher in energy there is the Mo Kβ_2_ emission line, which arises from a 4p to 1s transition. The final state of the Kβ_2_ decay process has a hole in the 4p shell, which interacts with the unpaired electrons in the 4d shell. Hence, the Kβ_2_ line for 4d TMs is analogous to the Kβ_1,3_ emission line for 3d TMs. However, 4d TM Kβ_2_ XES spectra do not exhibit well‐resolved splitting due in part to the smaller 4d–4p exchange integrals, relative to 3p–3d exchange integrals. In addition, the spectrum is further modulated relative to 3d TMs due to 1) the 4p^5^ final state SOC of a 4d TM being larger than the corresponding 3p^5^ final state SOC for 3d TMs and 2) the increased 1s core–hole lifetime broadening of 4d TMs relative to 3d TMs (1.2 eV for Fe and 4.52 eV for Mo).^49^

VtC XES results from electrons in primarily ligand‐based valence orbitals refilling the 1s core hole at the metal with the intensity due to the percent *n*p metal character mixed into the valence orbitals.^34^, ^37^ In 3d TMs, the Kβ′′ results from ligand ns to metal 1s transitions, while the Kβ_2,5_ results from ligand np to metal 1s transitions. In principle, analogous transitions should exist for the 4d TMs, and based on the energetics, these should be the Kβ′′ and Kβ_4_ transitions. Recently, Ravel et al. demonstrated that this analogy holds for the Kβ′′ features in niobium complexes,^25^ where the energy of the Kβ′′ features were shown to depend on ligand identity. In contrast, the Kβ_4_ feature remains relatively unexplored, and previous studies have assigned it as arising from orbitals possessing primarily metal 4d character, rather than a ligand *n*p to metal 1s transition.^27^ It is clear that a more detailed exploration as to the exact origin of the Kβ_4_ features is warranted.

Herein, we carry out a systematic study of molecular Mo complexes and examine how the local geometric and electronic structures impact the Mo Kβ_2_, Kβ′′, and Kβ_4_ features. The results are correlated to density functional theory (DFT) calculations in order to obtain more detailed insight. Complexes **0**–**VI** (Table 1, Figure 1) will first be presented to provide a detailed description of the Mo emission lines. We then utilize Mo Kβ XES together with Mo Kβ HERFD XAS to contrast the sensitivity of XES and XAS approaches for understanding electronic structural changes in two nitrogenase FeMoco related model complexes. The results allow for a better understanding of the origins of 4d TMs emission lines and also establish the utility of Mo XES for future applications in bioinorganic chemistry and catalysis.

## Results and Discussion

**Figure 3:**
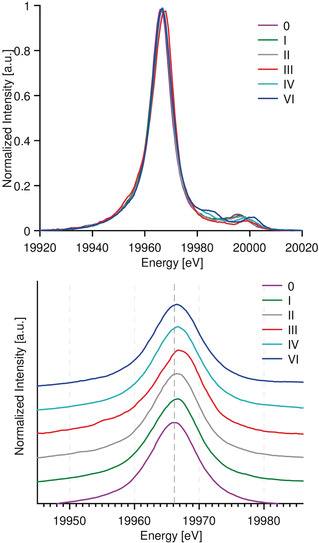
Mo Kβ XES spectra of compounds **0**–**VI** (top) and zoom of the Kβ2 region (bottom). Data presented with the intensity normalized to the Kβ2 maxima.

**Mo Kβ XES**. The normalized Mo Kβ XES spectra of compounds **0**–**VI** are presented in Figure 3 (top). The spectra consist of an intense Kβ_2_ feature at ≈19970 eV and weaker higher energy peaks in the range of 19 980–20 003 eV representing the VtC region (Kβ′′ and Kβ_4_).

**Kβ_2_ XES**. Figure 3 (bottom) shows an expansion of the Kβ_2_ feature. The Kβ_2_ spectra were fit with three pseudo‐Voigt function line shapes, where Peak 1 (Table 2) is the dominant curve and well‐represents the energy of the most intense feature. The vertical line at 19 966.1 eV in Figure 3 (bottom) corresponds to the energy position of **0** at the Kβ_2max_ (Fit Peak 1 in Table 2). The energy position of the Kβ_2max_ (Peak 1) is found within a narrow range from 19 966.1 and 19 966.6 eV for all compounds with the exception of (ttcn)MoCl_3,_ compound **III**, for which the Kβ_2max_ is at 19 967.2 eV. As the Kβ_2_ feature corresponds to a 4p to 1s transition with a 4p^5^4d^n^ final state, it will be influenced by 4p‐4d exchange coupling, and thus should be sensitive to the number of unpaired 4d electrons. The *S*=3/2 compound **III**, appears at highest energy due to the high‐spin d^3^ configuration.

**Table 2:** Interpretive pseudo‐Voigt fits of Mo Kβ XES with the corresponding position (Pos.) and Amplitude (Amp.) of the peaks.

Complex	Total	Kβ_2 max_		Kβ′′				Kβ_4_			
	spin	Peak 1		Peak 2		Peak 3		Peak 4		Peak 5	
	state	Pos. [eV]	Amp.	Pos. [eV]	Amp.	Pos. [eV]	Amp.	Pos. [eV]	Amp.	Pos. [eV]	Amp.
0	0	19 966.1	12.75	19 981.4	0.17	19 989.7	0.12	19 994.2	0.14	19 997.4	0.28
I	1/2	19 966.4	12.70	19 980.2	0.15	19 990.0	0.20	19 995.2	0.15	19 998.8	0.24
II	0	19 966.5	12.46	19 980.2	0.15	19 990.7	0.19	19 995.4	0.14	19 999.0	0.19
III	3/2	19 967.2	12.12	19 983.7	0.06	19 990.9	0.08	19 997.5	0.11	20 001.3	0.11
IV	1	19 966.6	13.16	19 981.5	0.22	19 988.7	0.13	19 997.2	0.22	20 001.6	0.14
VI	0	19 966.5	13.25	19 983.1	0.34	19 987.1	0.30	19 999.0	0.22	20 002.4	0.13

Using the isostructural models **0** and **I** for comparison, the energy of the Kβ_2max_ increases by ≈0.3 eV per unpaired electron for all *S*≤1 complexes. The apparent, albeit, small shifts in the Kβ_2_ emission energy as a function of Mo oxidation‐state clearly demonstrates the contribution of 4p–4d exchange coupling. However, this trend is significantly smaller than the 0.5–0.7 eV shift per unpaired electron shifts observed for 3d TMs.^50^, ^51^, ^52^ This is a result of the smaller 4p–4d exchange integrals relative to the corresponding 3p–3d integrals.^53^ We note that the shift will, of course, be further modulated by changes in the Mo 1s energy.

Although the shifts in the Kβ_2_ maxima are modest, the fits reveal an asymmetric profile in the Kβ_2_ feature (Table S1 and Figure S1), consistent with previous reports by Hoszowska et al.^28^ Due to the significant 1s core hole broadening for Mo, the position and intensity of the low energy shoulder is not well‐defined. However, we can discuss the possible origins of this asymmetry based on Russel Sanders terms in the atomic limit, in a similar fashion as previously done for 3d TM.

For 3d TMs, it is well‐established that the Kβ_1,3_ spectral shape, including the Kβ′ intensity, is dominated by the final state multiplet structure.^50^, ^52^, ^54^ More specifically, previous systematic studies of the contribution of the Slater–Condon parameters F^2,4^_dd_, F^2^_pd_ and G^1,3^_pd_ have shown that the p–d exchange integrals G^1,3^_pd_ dominate the spectral shape of 3d TMs. In order to assess whether or not a similar picture can be assumed for 4d TMs, we calculated the Kβ_1,3_ and Kβ_2_ XES spectra of Cr^3+^ and Mo^3+^, respectively (Figure S2), using ligand field multiplet calculations. The Slater–Condon parameters F^2,4^_dd_, F^2^_pd_ and G^1,3^_pd_ were individually varied between 100 and 50 % of their atomic values. The F^2^_pd_ and F^2,4^_dd_ parameters were shown to result in only minor perturbations for the Cr^3+^ spectra, and made essentially no contributions to the Mo^3+^ spectra. In contrast, the p–d exchange integral (G^1,3^_pd_) has a pronounced effect on the spectral shape for both Cr^3+^ and Mo^3+^. This agrees with previous interpretations of 3d TM XES,^49^ and indicates that Kβ_2_ spectra are dominated by 4p–4d exchange. We thus use this simple picture for further discussion of the spectral trends.

Examination of the fits reveals that compound **III** exhibits the greatest redistribution of intensity to lower energy, again consistent with increased 4p–4d exchange contributions for an *S*=3/2 complex relative to the *S*=0 to 1 complexes. This exchange interaction redistributes the multiplet intensity into two families of features, corresponding mainly to final state triplet and quintet states, as indicated in Table 3. The remaining compounds exhibit more comparable intensity distributions, consistent with the reduced d‐counts and the presence of π‐accepting carbonyl ligands in **0**, **I** and **II**, which results in delocalization of d‐character onto ligand π* orbitals.

**Table 3:** Russel Sanders Terms (2S+1*L*) for the XES process in compounds 0 to IV. All complexes are in a low spin configuration.

Comp	4d^*n*^	GS (1s^2^4d^n^)	IS (1s^1^4d^n^)	FS (1s^2^4p^5^4d^n^)
**0**	d^6^	^1^I (^1^A_1g_)	^2^I	^2^H, ^2^I,^2^K
**I**	d^5^	^2^I (^2^T_2g_)	^1,3^I	^1,3^H, ^1,3^I, ^1,3^K
**II**	d^4^	^3^H (^3^T_1g_)	^2,4^H	^2,4^G, ^2,4^H, ^2,4^I
**III**	d^3^	^4^F (^4^A_2g_)	^3,5^F	^3,5^D, ^3,5^F, ^3,5^G
**IV**	d^2^	^3^F (^3^T_1g_)	^2,4^F	^2,4^D, ^2,4^F, ^2,4^G

**Figure 4:**
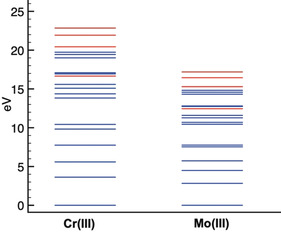
Calculated energy scheme for the allowed final states *n*p^5^ *n*d^3^ in the XES process (for Cr^3+^ vs. Mo^3+^: ^3,5^D, ^3,5^F, and ^3,5^G (color coded red for ^5^Γ states and blue for ^3^Γ states). Energy multiplet diagrams on different oxidation states included in the SI.

In order to understand the observed relatively modest changes in Mo Kβ_2_ compared to the reported effect of d‐count on the Kβ_1,3_ lines of 3d TMs, NEVPT2 CASSCF calculations were performed on Mo^3+^ and Cr^3+^ free ions. The XES final state 1s^2^ *n*p^5^ *n*d^n^ can be reached in the 1^st^ excited state of a p→d CASSCF calculation utilizing a 1s^2^ *n*p^6^ *n*d^*n*−1^ ground state. This first excited state will correspond to all multiplets allowed for *n*p^6^ *n*d^n−1^→*n*p^5^ *n*d^n^, from where only the multiplets that correspond to the final states XES processes were selected. The calculated XES final states (without SOC contributions) are shown in Figure 4, with the ^3^Γ states shown in blue and the ^5^Γ states shown in red. The CASSCF+NEVPT2 calculations for Group 6 Cr^3+^ and Mo^3+^ ions have the same dipole‐allowed XES final states. However, the final state XES multiplet energy distribution between these ions exhibits striking differences. In the case of Cr^3+^, the allowed XES final states span ≈23 eV, while for Mo^3+^ the final states span only ≈17 eV.

The decreased p–d exchange contribution to the Kβ_2_ spectra of 4d TMs relative to that observed in the Kβ_1,3_ spectra of 3d TMs is due to the increased delocalization of the 4d electrons. The inclusion of SOC in these calculations, and its influence on the 1s^2^ *n*p^5^ *n*d^3^ final states, are included in Figure S3A,B. The energy span of the final states remains almost the same in Cr^3+^ with a small growth of 0.06 eV, while there is a 1.5 eV increase in the energy expansion of the final states for Mo^3+^.

Hence, there is a total of 4.5 eV difference between the splitting of the final states for Cr^3+^ and Mo^3+^, of which only 20 % is due to spin–orbit coupling. The many calculated final state multiplets including SOC appear closer in energy and blur the ability to separate exchange‐split multiplets, contributing to the asymmetric profile found in Mo Kβ_2_.

A similar computational study was used to investigate the distribution of final states by varying the oxidation state. Figure S4 A,B shows an increase in the energy spread of the XES final states by ≈2.5 eV on going from Mo^1+^ (*S*=1/2) to Mo^2+^ (*S*=1) and by ≈5 eV on going from Mo^2+^ to Mo^3+^ (*S*=3/2). This suggests an expansion of ≈2.5 eV per unpaired electron in the purely ionic limit. Due to delocalization of d‐character onto the ligands, however, the effect is expected to diminish. This is clearly seen in the case of the carbonyl coordinated compounds where changes in spin state are effectively not observed at the Kβ_2_ line due to the strong π‐accepting nature of the ligand, as noted above and also previously observed in the Kβ_1,3_ mainline of 3d TMs.^55^

**Figure 5:**
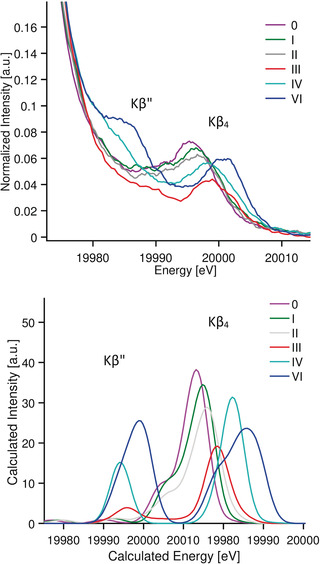
Experimental (Top) and calculated (bottom) VtC XES spectra for compounds **0**–**VI**.

**Mo valence‐to‐core Kβ_4_ and Kβ′′**. Resolved but far less intense Kβ_4_ and Kβ′′ transitions appear on the high‐energy tail of the Kβ_2_ mainline. Together, these transitions make up the Mo VtC region as shown in Figure 5 (top). If we assume that the classical 3d TMs VtC transition assignments are transferable to the Mo VtC, the following hypothetical assignments can be made:

1. The Kβ_4_ feature is primarily due to transitions from *n*p ligand valence orbitals to the metal 1s core hole. The intensity of this feature is dominated by the amount of metal p character mixed into these valence orbitals. The Kβ_4_ feature is the equivalent of the L(*n*p)→M(1s) Kβ_2,5_ feature for 3d TMs, where the intensities of the peak have been previously correlated with the M−L bond length
2. The Mo Kβ′′ peak is primarily due to transitions from *ns* ligand valence orbitals to the metal 1s core hole. It is dominated by ligand identity and the intensity is modulated by metal–ligand bond length.^27^

Experimental data were fit for all compounds, including the main peak positions and amplitudes, and are included in Table 2. The most intense peaks correspond to the Kβ_4_ features, located between 19 995–20 010 eV for compounds **0** to **VI**, with total areas of 0.2–0.4 as indicated in Table 2 (Peak 4 and Peak 5). The energy of the Kβ_4_ feature appears to track with oxidation state, with the feature increasing in energy by ≈5 eV on going from **0** to **VI**. This likely reflects, in large part, the stabilization of the Mo 1s energy upon increasing Z_eff_. Similar trends have been previously observed for 3d TM.^56^

As the weak Kβ′′ features appear on the high‐energy tail of the more intense Kβ_2_ mainline, the Kβ′′ features are either obscured or poorly resolved (between 19 975–19 990 eV, Peaks 2 and 3, Table 2). However, for the molybdenum oxides **IV** and **VI** relatively intense Kβ′′ features are observed.

**Figure 6:**
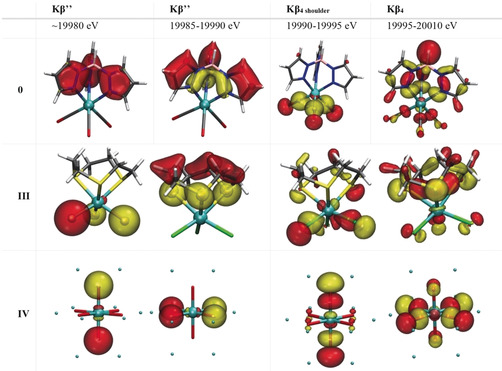
MOs involved in the most intense Kβ′′ and Kβ4 transitions for compounds **0**, **III**, and **IV**.

To further test the assignment of these transitions and gain additional insight into their molecular origin, the Mo VtC of each compound was calculated by ground‐state DFT methods.^34^ The calculated VtC spectra were obtained by allowing for electric dipole, magnetic dipole and quadrupole contributions. The resultant spectra were found to be comprised of 99.9 % dipole contributions. The DFT results well‐reproduce the experimental data, replicating the observed energy trends and transition intensities (Figure 5, bottom). The calculated spectra allow the donor molecular orbitals of each transition to be assigned. Figure 6 shows the primary contributing molecular orbitals (MO) for the Kβ′′ and the more intense Kβ_4_ transitions for compounds **0**, **III** and **IV**. A quantitative orbital analysis for all compounds is included in Table S2.

Qualitative inspection of the MOs contributing to the intensity of the Kβ_4_ peak demonstrate that this transition is mainly from *n*p ligand valence orbitals and that the calculated intensity is dominated by the amount of metal p character mixed into these valence orbitals. For instance, the most intense transition for **III** is in the Kβ_4_ region and is attributed to a Cl 3p → Mo 1s transition (≈19 999.4 eV with a total integrated area of 0.22 units Figure 6). The Kβ_4_ transitions for both **IV** and **VI** are from O 2p ligand MOs (Figure 5) with Mo 4p/5p dipole contributions. As **IV** and **VI** each have oxygen ligands, but different d electron counts, their comparison most directly tests the Kβ_4_ origin. The Kβ_4_ energy positions for **IV** and **VI** are ≈1.3 eV shifted and their total integrated areas are 0.36 and 0.33 units. It was previously suggested that the Kβ_4_ feature is formally a 4d → 1s transition.^27^ However, complex **VI** is formally d^0^ with no available donor d‐electrons for the Kβ_4_ transition. Therefore, this further supports the assignment of the Kβ_4_ feature as a ligand *n*p → Mo 1s transition, which gains intensity through metal *n*p character mixing into the ligand orbitals. Only complexes **III**, **IV**, and **VI** have well‐resolved Kβ′′ features. The MOs which correspond to these transitions are ligand *ns* based. The calculated spectrum for complex **III** shows one main feature due to the Cl 3s → Mo 1s transition between 19 987–19 990 eV and contributions from the ttcn ligand, found at 19 990 eV but with low intensity. Calculated XES spectra of **IV** and **VI** show one main Kβ′′ feature due mainly to O 2s‐based molecular orbitals. These transitions possess slightly different intensities due to varying Mo−O distances. Complex **VI**, MoO_3_, has an orthorhombic crystal distorted bulk unit with strong anisotropy in Mo−O bonding that is also responsible for a higher dipole contribution relative to the monoclinic MoO_2_ complex **IV**. The shortest Mo−O bond distance in MoO_3_ is 1.67 Å while the shortest Mo−O bond length for MoO_2_ is 2.023 Å. These calculations validate the aforementioned hypothesized transition assignments (see above).

The back‐bonding character of the carbonyl ligands yields VtC transitions that are more mixed in character. The rich Fe VtC spectra of Fe carbonyl complexes exhibit features arising from σ* 2s–2s (CO) and *σ* 2p_*z*_–2p_*z*_ (CO) interactions with the metal.^55^ For the Mo carbonyl‐containing compounds, well‐resolved contributions from the carbonyl ligands are not observed. However, the calculations reproduce experimental trends, with the energy of the Kβ_4_ increasing and the intensity decreasing going from **0** to **II**. The σ‐type bonding interactions are capable of mixing with Mo p orbitals to form available donor orbitals that are major contributors to the Kβ_4_ region, Figure 6. The transitions in the Kβ_4_ region with the highest dipole contribution to the emissionoscillator strength correspond to transitions from *σ* 2p_*z*_‐2p_*z*_(CO) + σ N(Tp) MOs. The lessening of π back‐donation from the Mo 4d shell is reflected in Mo‐CO bond lengthening from **0** to **II**. The increase in Mo−CO bond length affects the intensity of VtC features as the *n*p Mo‐ligand mixing decreases. This same distance dependence is observed in the Kβ_2,5_ feature of 3d TMs.^34^, ^37^

For compounds **0** to **II**, three distinguishable peaks are found to be part of the Kβ′′ calculated transitions. The two transitions between 19 980–19 985 eV are of significantly lower intensity than the other calculated Kβ′′ features and not observed in the experimental data. The low intensity features are due to N and C 2s orbital delocalization over the tris(pyrazolyl)borate (Tp) ligand. From an MO description, both features can be assigned as transitions from MOs of the chelating pyrazole rings in the Tp ligand with little contribution of the Mo(CO)_3_ fragment. The minimal amount of metal p character mixed into these MOs results in very small dipole contributions to the calculated oscillator strength. This explains why these transitions are not observed in the experimental spectra.

Despite the disparate ligand character of the studied Mo complexes, clear trends in the Mo VtC emerge: the Kβ_4_ feature provides a marker of Mo oxidation‐state and is sensitive to ligand identity and metal ligand bond length.

**Figure 7:**
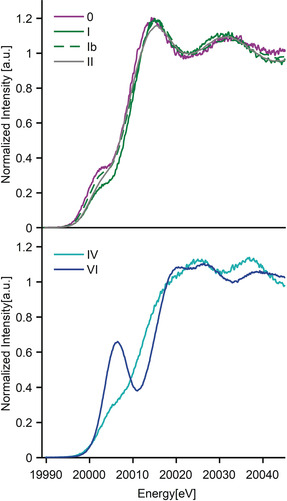
Kβ_1_ HERFD XAS spectra of Mo compounds containing CO ligands **0** to **II** (top) and Mo oxides **IV** and **VI** (bottom).

**XAS measurements**. Figure 7 displays the Mo Kβ_1_ HERFD XAS of the complexes grouped by ligand identity, with the Mo compounds with Tp and CO ligands (**0, I**, **Ib** and **II**) at the top and the oxides (**IV** and **VI**) at the bottom. Metal K‐edge positions shift towards higher energies with increasing oxidation state, as the effective nuclear charge varies.^57^, ^58^ Figure 7 (bottom) shows an energy shift of +2.6 eV in the rising edge on going from compound **IV** to **VI**. Figure 7 (top) shows a ≈1 eV energy shift at the white lines, consistent with the increase in oxidation state from **0** → **I** → **II**. However, identification of the Mo oxidation state of these compounds by the inflection points of their corresponding rising edges has a less intuitive interpretation. In this particular case, the rising edge of each compound is modulated by the strong back‐bonding nature of the carbonyl ligands, with an increase in back‐bonding resulting in an edge shift toward higher energy. Interestingly, for the present series, the Kβ_4_ XES features seem to more clearly correlate with the oxidation states of the compounds than the rising edge positions.^56^

**A practical case: molybdenum–iron–sulfur clusters**. Having established in the preceding sections the complementary information that can be obtained by the use Mo Kβ XES and Kβ_1_ HERFD XAS, we now go on to utilize these methods to understand synthetic MoFe cubanes.^59^ These serve as examples of more complex models with relevance to a biological system, in this particular case the FeMoco active site of nitrogenase. Two MoFe cubanes,^59^ [MoFe_3_S_4_]^3+^ and [MoFe_3_S_4_]^2+^, were studied to determine the sensitivity of each method to one electron cluster reductions. In addition, Fe Kβ_1,3_ HERFD experiments are also presented in order to obtain insight into the location of the redox event.

**Figure 8:**
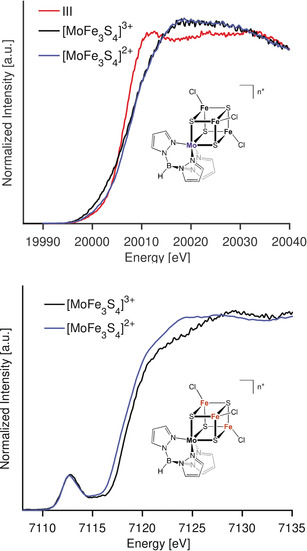
Mo Kβ_1_ (top) and Fe Kβ_1,3_ (bottom) HERFD XAS spectra of **III** (Mo only), [MoFe_3_S_4_]^3+^ and [MoFe_3_S_4_]^2+^.

Figure 8 displays both Mo and Fe Kβ HERFD‐XAS spectra. The Fe Kβ_1,3_ HERFD‐XAS in Figure 8 (bottom) shows a 0.7 eV decrease in the rising edge position on going from [MoFe_3_S_4_]^3+^ to [MoFe_3_S_4_]^2+^, indicating an iron‐based reduction. Figure 8 (top) shows (ttcn)MoCl_3_ compound **III** and both MoFe cubanes. While all three complexes contain a Mo^3+^ atom, complex **III** clearly is very different in edge shape and whiteline position. This may be attributed to the different ligation environment and the differences in local Mo spin state (*S*=3/2 for **III** vs. *S*_loc_(Mo)=1/2 for the cubanes).^60^, ^61^ Both MoFe cubanes overlap in the whiteline region of the Mo K‐edge XAS spectra, suggesting there is no change in oxidation state at the Mo site. This is consistent with reduction occurring at the iron, as supported by the Fe K‐edge data and previous Mössbauer studies.^62^ Just before the rising edge, both compounds **III** and [MoFe_3_S_4_]^2+^ overlap in the pre‐edge area, while [MoFe_3_S_4_]^3+^ has a higher intensity pre‐edge. This may reflect greater p–d mixing in the [MoFe_3_S_4_]^3+^ cubane due to increased covalency and/or the presence of MMCT transitions.

**Figure 9:**
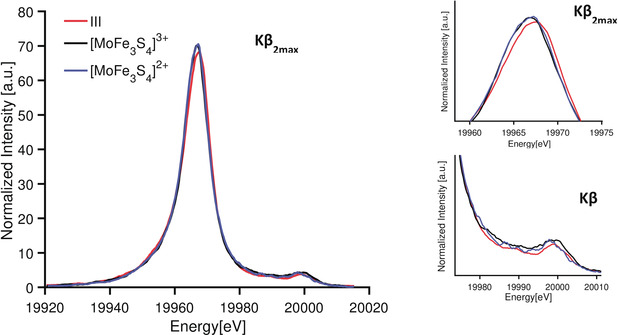
Mo Kβ_2_ and Kβ_4_ XES spectra of compounds **III**, [MoFe_3_S_4_]^3+^, and [MoFe_3_S_4_]^2+^. Plots at the right side contain both regions enlarged.

Figure 9 displays the Mo Kβ_2_ and VtC for **III** and both MoFe cubanes. The Kβ_2max_ of compound **III** (*S*=3/2) is found at 19 967.2 eV while both MoFe cubanes (*S*_loc_(Mo)=1/2) show their Kβ_2max_≈0.6 eV downshifted from the Kβ_2max_ of **III**. This translates to a 0.3 eV energy shift per unpaired electron in Mo Kβ_2max_, and is fully consistent with the shifts determined for compounds **0**–**VI**. We note, however, that the shift may also be attributed to an increase in covalency upon replacing the thioethers in **III** with the more covalent sulfides in the cubane clusters.^50^

Figure 9 (lower right) shows the enlarged Kβ_4max_ region for all three complexes. [MoFe_3_S_4_]^2+^ has a Kβ_4_ maximum at 19 998 eV, about 1 eV lower in energy than the [MoFe_3_S_4_]^3+^ and (ttcn)MoCl_3_ Kβ_4max._ Although MoFe cubanes share the same oxidation and spin state at the Mo site, differences in the Kβ_4_ features suggest additional electronic structural contributions to this spectral region.

**Figure 10:**
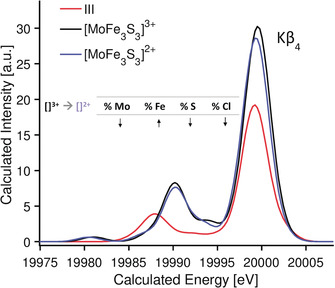
DFT XES calculated spectra of compounds **III**, [MoFe_3_S_4_]^3+^, and [MoFe_3_S_4_]^2+^.

**Figure 11:**
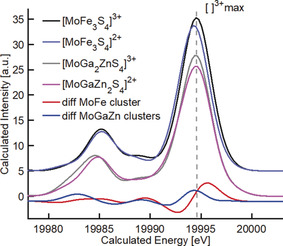
DFT XES calculated spectra of [MoFe_3_S_4_]^3+^ and MoFe_3_S_4_]^2+^, the hypothetical cubanes: [MoGa_2_ZnS_4_]^3+^ and [MoGaZn_2_S_4_]^2+^, and their corresponding difference spectra ([ ]^3+^–[ ]^2+^) for the MoFe) and the MoGaZn clusters. All spectra were aligned to the maximum of [MoFe_3_S_4_]^3+^.

XES spectra were calculated by DFT in order to understand the origin of these differences (Figure 10, Figure 11). In both experimental and calculated spectra, compound **III** shows a Kβ_4_ peak with lower intensity than the MoFe cubanes. In **III**, the intensity of this peak is mainly due to transitions from 3p Cl and thioether sulfur MOs (Figure 6). However, for both MoFe cubanes the origin of the Kβ_4_ transitions is less straight forward as the calculated transitions are modulated by covalency (via Mo−S(thiolate) and Mo−Fe bonding). For the MoFe cubanes, the Mo−N(Tp) and Mo−S average distances decrease by 0.03 eV and 0.02 eV, respectively, going from [MoFe_3_S_4_]^2+^ to [MoFe_3_S_4_]^3+^. Furthermore, the MOs involved in the Kβ_4_ transitions show a decrease of 0.9 % in Mo *n*p character and an increase of 4 % in Fe 3d character.

The putative effect of metal–ligand bonding on the XES VtC spectra of both cubanes was investigated by replacing the Fe sites with closed shell atoms without changing overall geometry or local Mo spin. Figure 11 shows the DFT calculated XES spectra for [MoFe_3_S_4_]^3+^, [MoFe_3_S_4_]^2+^ and their corresponding MoGaZn substituted cubanes: [MoGa_2_ZnS_4_]^3+^ and [MoGaZn_2_S_4_]^2+^ (with a Mo local spin of 1/2 ). MoGaZn cubanes are hypothetical models that use MoFe cubane structure without computing optimization geometries. Comparisons with the real MoFe cubanes were made by shifting in energy the calculated MoGaZn spectra to align both [MoGa_2_ZnS_4_]^3+^ and [MoFe_3_S_4_]^3+^. Unlike the XES computed for the MoFe cubanes, the calculated energy position at the Kβ_4_ maximum remains the same for both MoGaZn cubanes. These results demonstrate that the experimental Mo VtC XES of the MoFe cubanes is sensitive to the reduction occurring at the Fe site.

Since the ground states for [MoGa_2_ZnS_4_]^3+^ and [MoGaZn_2_S_4_]^2+^ have Mo local spins of 3/2, XES spectra were also calculated for the *S*_loc_(Mo)=1/2 spin states. No energy shift was observed in the Kβ_4_ peak position and the Kβ_4_ intensity remained higher for the [MoGa_2_ZnS_4_]^3+^ cubane (Figure S6).

This computational study demonstrates that Mo VtC is moderately sensitive to metal–metal interactions, but relatively insensitive to the Mo local spin state.

## Conclusion

A systematic experimental and theoretical study of Mo Kβ XES has been performed for a range of molecular and extended lattice Mo materials. The ability of this method to provide local information on Mo oxidation state, spin state, and identity of coordinated ligands has been established. The trends elucidated here should be readily transferable to other 4d TMs.

Having established the strong correlations between experimental and calculated Mo Kβ XES spectra, the method was extended to MoFe_3_ cubane clusters with relevance to the FeMo cofactor of nitrogenase. These studies highlight the ability of Mo Kβ XES to report on subtle changes in cubane electronic structure. By combining the XES studies with XAS measurements at the Mo and Fe K‐edge, it is demonstrated that the Mo Kβ XES is sufficiently sensitive to show changes upon iron‐based reduction. This suggests that Mo Kβ XES could be useful for studies of FeMoco and might provide greater selectivity than Fe Kβ XES, where contributions from the 7 iron of FeMoco and the 8 irons of the P‐cluster greatly complicate the spectral interpretation.

The present study forms the basis for applying Mo Kβ XES in homogeneous and heterogenous catalysis. While Mo Kβ XES suffers from relatively large Mo 1s core–hole lifetime broadening,^22^, ^27^ this technique may be particularly useful for Mo‐containing enzymes with a large number of S atoms present. Due to the overlap of the Mo L‐edges and S K‐edges, it is difficult to deconvolute the Mo and S contributions in the tender X‐ray spectra.^63^

The approach presented here may provide a means to more readily access electronic structural changes at the Mo in biological systems where molybdenum is involved either as a mononuclear active site (DMSO reductase, sulfite oxidase, etc) or as part of multinuclear metal centres, like Cu,Mo‐containing CO dehydrogenases.^64^

## Conflict of interest

The authors declare no conflict of interest.

## Supporting information

As a service to our authors and readers, this journal provides supporting information supplied by the authors. Such materials are peer reviewed and may be re‐organized for online delivery, but are not copy‐edited or typeset. Technical support issues arising from supporting information (other than missing files) should be addressed to the authors.

Supplementary
