# Supplementary material for: Kβ X‐Ray Emission Spectroscopic Study of a Second‐Row Transition Metal (Mo) and Its Application to Nitrogenase‐Related Model Complexes
Source: Angew Chem Int Ed Engl. 2020 May 29;59(31):12965–75. doi: 10.1002/anie.202003621 (PMC7496169; doi:10.1002/anie.202003621)
Supplement: Supplementary file 1 — Supplementary [file ANIE-59-12965-s001.pdf]

Angewandte Chemie
Eine Zeitschrift der Gesellschaft Deutscher Chemiker

Supporting Information

Kβ X-Ray Emission Spectroscopic Study of a Second-Row Transition Metal (Mo) and Its Application to Nitrogenase-Related Model Complexes

*Rebeca G. Castillo, Justin T. Henthorn, Jeremy McGale, Dimitrios Maganas, and Serena DeBeer**

anie_202003621_sm_miscellaneous_information.pdf

Supporting Information

Table of Contents

EXPERIMENTAL METHODS 1

SAMPLE PREPARATION

DATA COLLECTION

COMPUTATIONAL STUDIES

TABLE S1 &FIGURE S1 3

FIGURE S2 4

FIGURE S3 5

FIGURE S4 6

TABLE S2 7

FIGURE S5 8

FIGURE S6 9

FIGURE S7 10

REFERENCES 11

Experimental Methods

Sample preparation. Compounds $[TpMo(CO)_3][Et_4N]$ (Tp=hydridotris(pyrazoly)borate) **(0)**[1] , $[TpMo(CO)_3]$ **(I),**[2] $[Tp_2Mo_2(CO)_4]$ **(Ib),**[2] $[Tp_2Mo_2(CO)_4](\mu\text{-}S)$ **(II),**[3] and (ttcn)$MoCl_3$ (ttcn=1,4,7-trithiacyclononane) **(III)**[4] were synthesized following published procedures. MoO_2 and MoO_3 were purchased from Sigma and used as received. Sample preparation for X-ray measurements was achieved by grinding the solid compounds and diluting with BN to minimize self-absorption effects during the HERFD XAS measurements. Both synthetic cubane cluster models $(Et_4N)[(Tp)MoFe_3S_4Cl_3]$ ($[MoFe_3S_4]^{3+}$) and $(Et_4N)[(Tp)MoFe_3S_4Cl_3]$ ($[MoFe_3S_4]^{2+}$) were synthesized as previously reported.[5] Separate samples were prepared for Fe and Mo measurements with different dilution ratios according to the different X-ray absorption cross sections.

Data Collection. All presented data were measured at the ID26 beamline of the European Synchrotron Radiation Facility (ESRF) with the storage ring operating at 6 GeV and injection currents of 90 mA in a 16-bunch filling mode. Si(311) and Si(111) double-crystals monochromators were used upstream for energy selection for the Fe and Mo experiments, respectively. The monochromatic incident energy was calibrated to the fist inflection point of a Fe foil set to 7111.2 eV and the maximum of the white line of a Mo foil set to 20016.4 eV. The beam-size was 500x100 microns^2 providing a flux density of ~1x10^{12}ph/s. Mo X-ray absorption spectra were measured simultaneously in total fluorescence yield (TFY) and $K\beta_1$ HERFD-detection modes. For the emission measurements, a 1 m radius Johann-type XES spectrometer was used, equipped with four or five spherically bent Ge (620) and Si (12 12 0) analyzer crystals for Fe and Mo experiments, respectively. The nominal analyzer Bragg angle for Fe $K\beta_{1,3}$ was 73.1° and 76.03° for Mo XES $K\beta_1$. The XES spectrometer was internally calibrated using the emission lines of Fe_2O_3 ($K\beta_{1,3}$ = 7059.4 eV; Kβ' = 7044.9 eV) and MoO_2 ($K\beta_1$ = 19607.8 eV; $K\beta_2$ = 19966.6 eV). Kβ-detected XAS and Kβ XES data collection was done by using a dead-time corrected silicon drift diode detector (Ketek), aligned on the Rowland circle. Possible attenuation of the fluorescence signal was reduced by placing a He-filled flight path between the sample, the analyzer crystals and detector. The total energy resolution was estimated to be approximately 1.2 eV for Fe measurements and ~5 eV for the Mo experiment, based on the FWHM of the elastic peaks. The width of the elastic peak (ΔE) results from a convolution of the spectrometer and monochromator resolution.

Mo $K\beta_1$-HERFD XAS was collected over an energy range of 19990 - 20090 eV in 0.2 eV steps, and longer scans where collected for normalization procedures within an energy range of 199117 – 207107 eV in 0.6. eV steps.

The incident energy was set to 20200 eV to collect non-resonant Mo XES. Emission scans were collected varying the scan parameters in 3 different rages: 199200-19963 eV, 19963-19986 eV, and 19986-20020, using an energy step sizes of 0.4 eV, 0.35 eV, and 0.25 eV, respectively.

Radiation damage studies were done on each individual sample by collecting successive fast energy range XAS scans (10 sec/scan) at a single spot on the sample, using multiple spots. Maximum dwell time per spot to collect undamaged data on all samples was 40-120 seconds. The present data comprises an average of ~10 XAS scans and ~4-8 XES scans on each compound. Total collection time for XES measurements was ~20 minutes per sample. In order to test the feasibility for future protein measurement, preliminary data on ~300 uM resting MoFe nitrogenase were obtained. A Mo $K\beta_1$-HERFD XAS scan gave a signal of ~110 counts/sec (compared to ~15,000 counts/sec for the measured model complexes), indicating more diluted samples will require significantly increased acquisition times, but should still be feasible with a dedicated beam time. At ~300 uM Mo concentration, we estimate 45 hours of data collection will be needed. At concentrations of ~1 mM Mo, ~15 hours would be needed for high quality protein data. This indicates that future applications to proteins will be challenging, but certainly feasible.

Data Reduction. Individual scans showing no evidence of radiation damage were first averaged with the PyMCA[6] software package. XAS data processing was done using MATLAB, employing additional functions supplied in EasySpin 5.0.2.[7] A quadratic polynomial function was used to fit the post edge, followed by a normalization of the edge jump to one.

XES spectra were normalized to the maximum value of the $K\beta_2$ feature. The intensities and energy positions in XES data were extracted by modelling the experimental line shapes with Pseudo-Voigt functions and least-squared minimization based on the Levenberg-Marquardt algorithm, included in Python 3.

Computational studies. All calculations were performed using the ORCA[8] quantum chemistry package, version 4.1, developed by Neese and coworkers. Geometry optimizations were performed by using XRD structures as starting points. The TPSSh hybrid functional, basis set def2-TZVP and auxiliary basis sets sarc/j, previously reported to give correct ground state solutions for Mo-containing model compounds,[9] were employed. Computational core-level spectroscopy for XES was carried out by a ground-state DFT procedure, where transition energies are based on energy differences between one-electron Kohn-Sham orbitals, as previously reported.[10] MoO_2 and MoO_3 calculations required consideration of infinite lattice effects and the use of embedded clusters.[11-13] Capped effective core potentials were used to replace the metal and oxygen atoms. The embedded cluster calculations were done by using the xyz file of a Mo atom center extended to a radius of 100 Å, and a reduced embedding model was generated where point charges extend to 40 Å from the absorber. A quantum cluster was defined by 7 atoms and a boundary region (BR) of 10-30 atoms was used to study size effects on the spectra.

NEVPT2 CASSCF[14] calculations on two open shell systems (p and d shell) were performed on Mo and Cr ions to evaluate the influence of *n*p-*n*d exchange interactions, as well as to evaluate the effect of spin-orbit coupling within the final states. The XES final state $1s^2np^5nd^n$ can be reached in the 1st excited state of a p→d CASSCF calculation utilizing a ground state $1s^2np^6nd^{n-1}$. This first excited state will correspond to all multiplets allowed for $np^6nd^{n-1}\rightarrow np^5nd^n$, from where only the multiplets that correspond to the final states XES processed were selected.

For evaluating the case of free ions $3d^3$ vs $4d^3$, Cr^{3+} and Mo^{3+} were studied. Both contain three electrons in their 3d and 4d shells, respectively, thus they share the same CASSCF ground state $1s^2 np^6 nd^2$, as well as the same number of dipole allowed $1s^2 np^5 nd^3$ XES final states (^{3,5}D,F,G).

All LFT calculations were performed by using a pre-released version of the orca_lft functionality, a multiplet program which is part of the ORCA code. In order to investigate the effect of the Slater-Condon Parameters individually, we also simulated the XES spectra of the Cr^{3+} and Mo^{3+}. To this end, the core-valence 1s→3/4d electron excitations, as well as the inner shell valence to core 3/4 p→1s electron decays, had to be considered to create a 3-shell SPD. However, as one deals with core ionized states, it is possible to separate the problem into a 2-shell individual LFT problems, an SD LFT that reflects the core excitation process and a PD LFT problem that reflects the electron decay process. Hence this can be thought of as an effective 3-shell SPD problem in which the SP shell interaction is neglected.

The Slater-Condon parameters are provided in the SD and PD 2-shell based on ab initio ligand theory (AILFT)15 calculations at the CASSCF level. These parameters can then be utilized to simulate the XES spectra and tuned individually.

In all computed spectra, a constant broadening was employed via the orca_mapspc utility. This broadening was chosen to be 3 eV for Cr and 10.5 eV for Mo. The utilized broadenings were chosen to obtain reasonable agreement with the experimental spectra and the trends are consistent with the differences in 1s core-hole lifetime broadening (Γ_{3d}~ 1-1.3 eV, Γ_{4d}~ 4.5 eV).

Table S1. Representative fits of Mo model complexes as explained in the Experimental Section of this SI.

	$K\beta_2$				$K\beta''$		$K\beta_4$					
	Peak 0		Peak 1		Peak2		Peak3		Peak4		Peak5	
	Pos (eV)	Amp.	Pos (eV)	Amp.	Pos (eV)	Amp.	Pos (eV)	Amp.	Pos (eV)	Amp.	Pos (eV)	Amp.
**0**	19954.52	0.81	19966.07	12.75	19981.4	0.17	19989.70	0.12	19994.22	0.14	19997.4	0.28
**I**	19955.00	0.85	19966.33	12.70	19980.20	0.16	19989.97	0.23	19995.22	0.15	19998.80	0.24
**II**	19955	0.88	19966.46	12.46	19980.20	0.15	19990.70	0.19	19995.44	0.14	19999	0.19
**III**	19957.0	1.81	19967.23	12.12	19983.7	0.06	19990.89	0.08	19997.50	0.11	20001.33	0.11
**IV**	19955.0	0.54	19966.59	13.16	19981.54	0.22	19988.75	0.13	19997.20	0.22	20001.59	0.14
**VI**	19954.54	0.47	19966.5	13.25	19983.08	0.34	19987.11	0.30	19999.0	0.22	20002.41	0.13

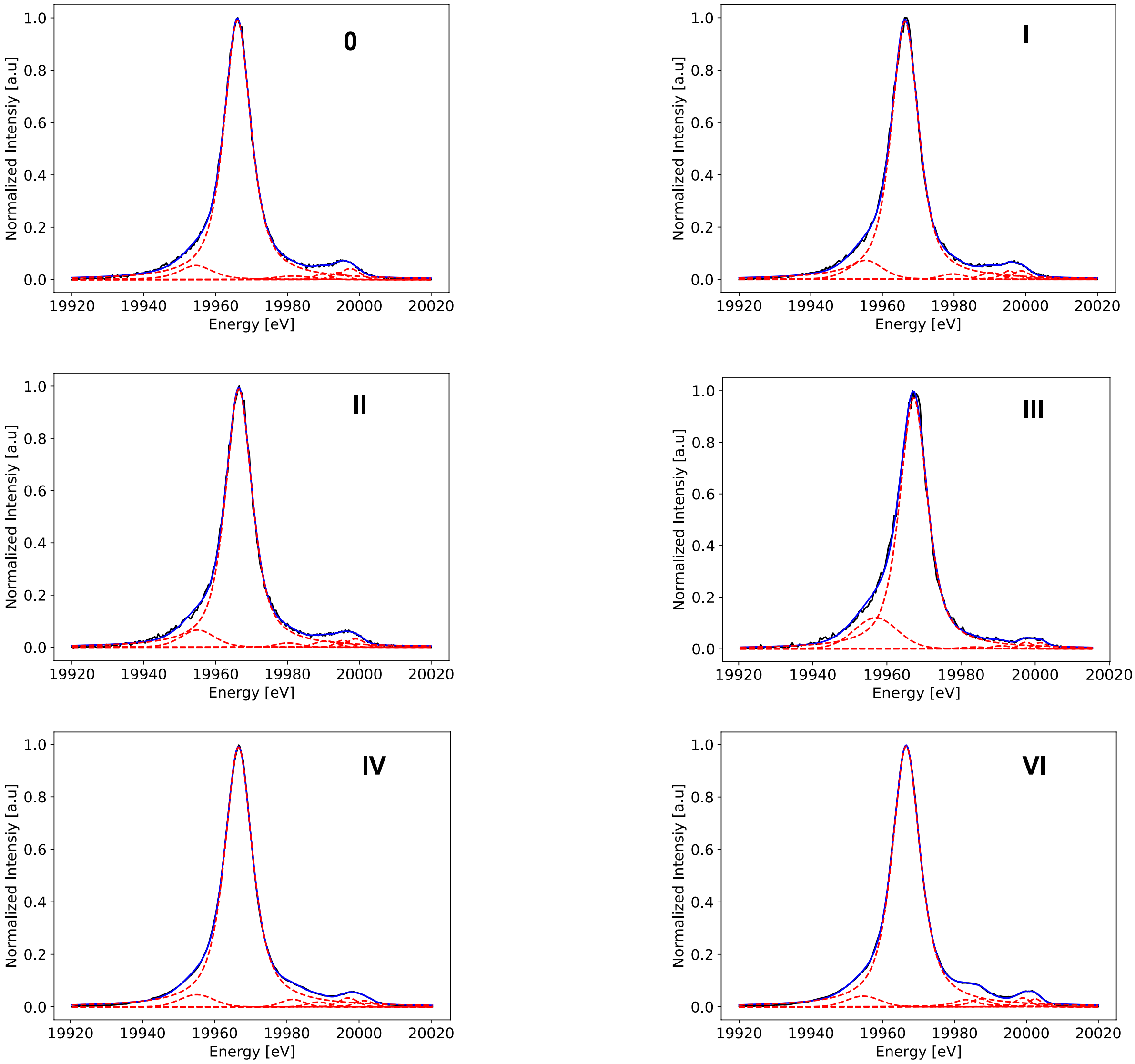

Figure S1. Representative fits of Compounds **0**-**VI**.

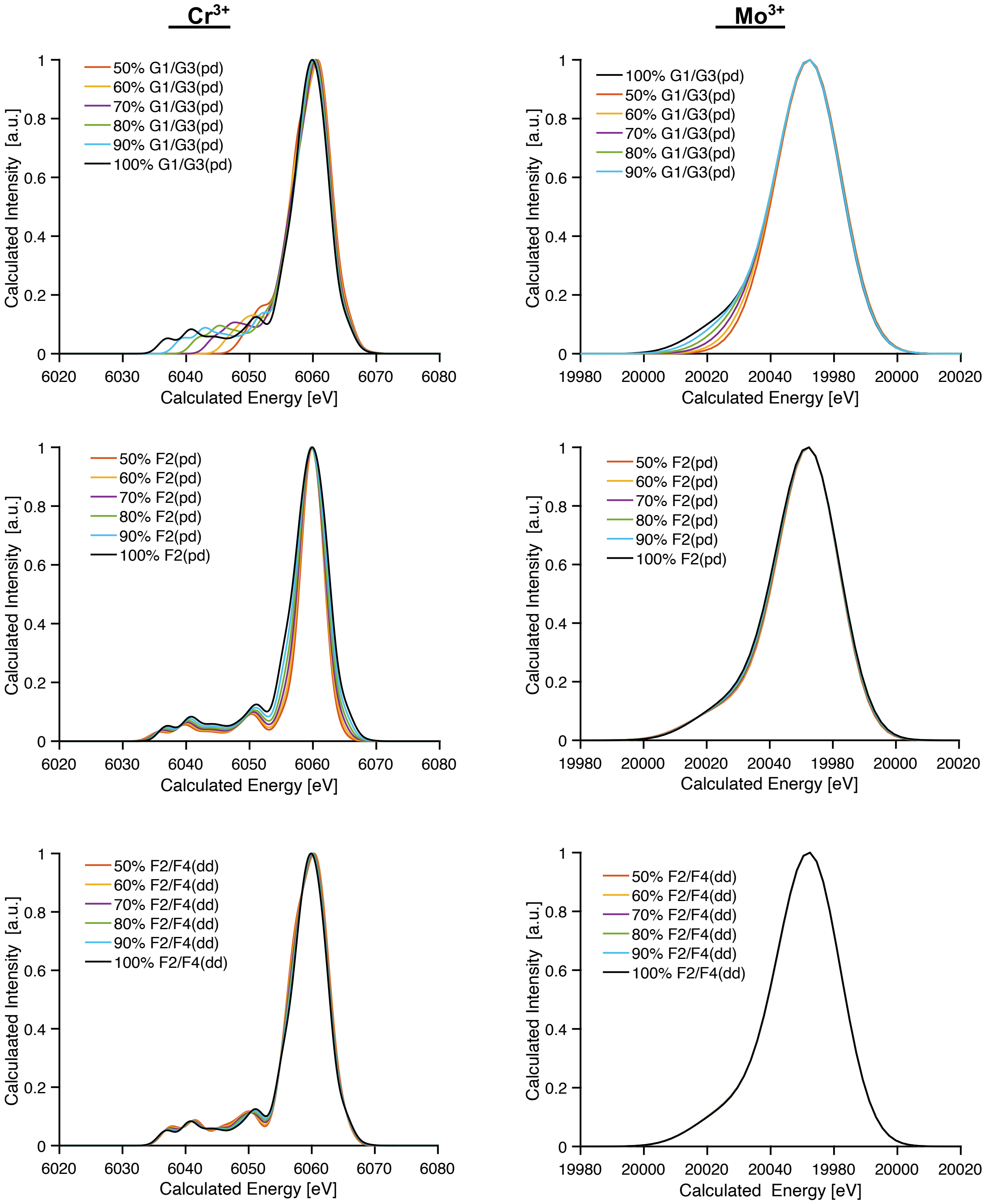

Figure S2. Calculated XES spectra for Cr^{3+} (left) and Mo^{3+} (right) showing the effect of scaling the Slater-Condon Parameters (exchange integrals, $G^{1,3}_{pd}$, top; Coulomb integrals, F^{2}_{pd}, middle; and electron repulsion, $F^{2,4}_{dd}$, bottom) from 50-100% of their atomic values. The calculated spectra are normalized to the maxima.

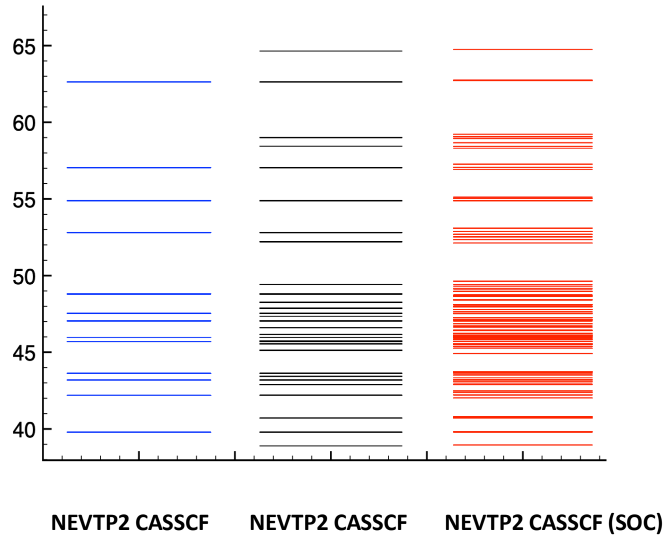

Figure S3A. Calculated energy scheme for the XES allowed $4p^5 4d^3$ final states for a Mo^{3+} atom limited to ^{3,5}D,F,G states (blue), all NEVPT2 CASSCF computed states (black) and all NEVPT2 CASSCF computed including spin-orbit coupling contributions (red).

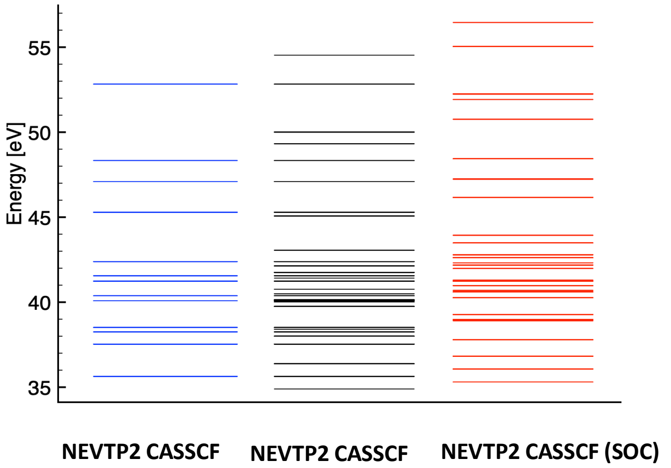

Figure S3B. Calculated energy scheme for the XES allowed final states $3p^5 3d^3$ for a Cr^{3+} atom limited to ^{3,5}D,F,G states (blue), all NEVPT2 CASSCF computed states (black) and all NEVPT2 CASSCF computed including spin-orbit coupling contributions (red).

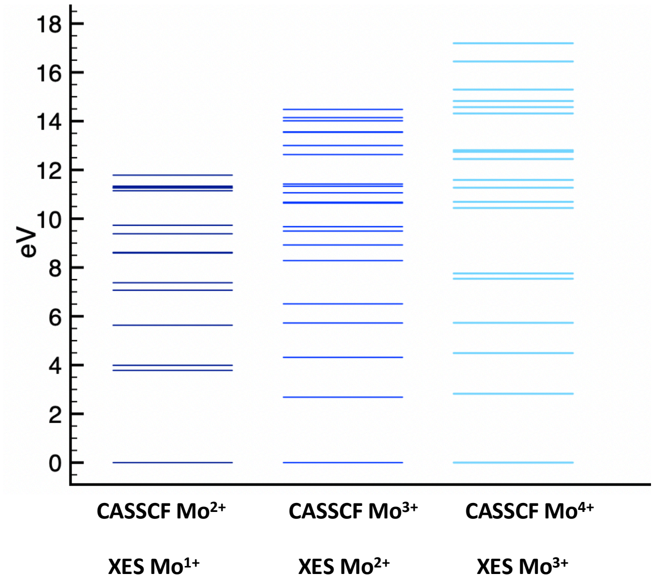

Figure S4A.Calculated energy scheme for the allowed XES final states for Mo^{1+}, Mo^{2+} and Mo^{3+} atoms. Figure displayed as XES Energy gap with the lower energy transition aligned to zero.

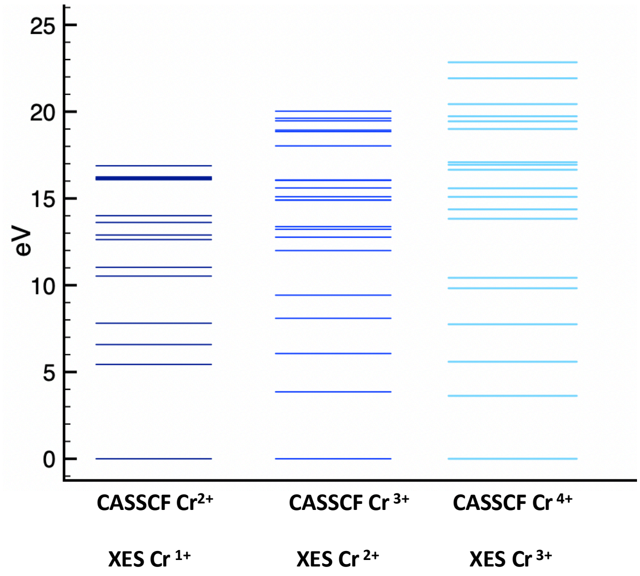

Figure S4B. Calculated energy scheme for the allowed XES final states for Cr^{1+}, Cr^{2+} and Cr^{3+} atoms. Figure displayed as XES Energy gap with the lower energy transition aligned to zero.

Table S2. Analysis of the primary contributing molecular orbitals (MO) for the Kβ" and $K\beta_4$ transitions. Each block in the table corresponds to the MO contributing the greatest intensity in the specified energy range.

		**Kβ''**			**Kβ''**			**$K\beta_4$ shoulder**			**$K\beta_4$**		
		**~19980 eV**			**19985-19990 eV**			**19990-19995 eV**			**19995-20010 eV**		
**0**		**% *n*s**	**% *n*p**	**% nd**	**% ns**	**% *n*p**	**% nd**	**% ns**	**% *n*p**	**% nd**	**% ns**	**% *n*p**	**% nd**
	**Mo**	0.7	2.0	0.0	0.3	0.5	0.0	2.6	2.5	0.0	0.0	7.6	0.8
	**CO Ligand**	0.0	0.0	0.0	0.0	0.0	0.0	34.6	51.4	2.7	6.6	11.6	0.6
	**Tp Ligand**	66.5	25.8	4.6	52.5	41.0	5.7	2.0	4.1	0.0	8.0	58.4	6.4
**I**		**% *n*s**	**% np**	**% nd**	**% ns**	**% np**	**% nd**	**% ns**	**% np**	**% nd**	**% ns**	**% np**	**% nd**
	**Mo**	0.8	1.9	0.0	0.4	0.5	0.1	0.5	2.8	0.3	0.4	7.7	0.9
	**CO Ligand**	0.0	0.0	0.0	0.0	0.0	0.0	22.1	34.4	1.3	4.4	8.0	0.2
	**Tp Ligand**	66.1	25.6	5.2	51.6	41.0	6.3	14.0	24.2	0.4	9.7	62.5	5.8
**II**		**% *n*s**	**% np**	**% nd**	**% ns**	**% np**	**% nd**	**% ns**	**% np**	**% nd**	**% ns**	**% np**	**% nd**
	**Mo**	0.8	1.9	0.1	0.4	0.5	0.0	0.3	1.7	3.1	0.1	7.3	3.3
	**μ-S**	0.0	0.0	0.0	0.0	0.0	0.0	0.1	0.0	0.0	0.1	1.8	0.0
	**CO Ligand**	0.0	0.0	0.0	0.0	0.0	0.0	22.5	35.4	1.3	3.9	5.5	0.0
	**Tp Ligand**	66.6	26.4	4.1	51.7	41.4	6.0	12.1	23.1	0.4	9.3	63.2	5.4
**III**		**% *n*s**	**% np**	**% nd**	**% ns**	**% np**	**% nd**	**% ns**	**% np**	**% nd**	**% ns**	**% np**	**% nd**
	**Mo**	0.0	8.4	6.3	1.0	1.6	0.0	0.0	5.1	7.6	0.6	5.4	3.7
	**Cl Ligand**	84.1	0.0	0.0	0.9	0.0	0.0	1.2	56.4	0.1	0.0	17.7	0.0
	**Tp Ligand**	0.8	0.4	0.0	55.5	36.4	4.6	5.5	22.0	2.1	9.2	57.0	6.4
**IV**		**% *n*s**	**% np**	**% nd**	**% ns**	**% np**	**% nd**	**% ns**	**% np**	**% nd**	**% ns**	**% np**	**% nd**
	**Mo**	0.0	10.2	0.0	0.0	11.9	0.0	0.0	4.0	0.0	0.0	3.8	0.0
	**O**	88.5	1.0	0.0	87.6	0.4	0.0	1.2	94.8	0.0	0.9	95.3	0.0
**VI**		**% *n*s**	**% np**	**% nd**	**% ns**	**% np**	**% nd**	**% ns**	**% np**	**% nd**	**% ns**	**% np**	**% nd**
	**Mo**	3.3	5.3	8.5	2.4	8.1	2.5	0.9	2.9	5.5	0.3	2.4	2.4
	**O**	81.4	1.2	0.0	78.9	0.8	0.0	4.4	85.5	5.6	1.4	93.3	0.0

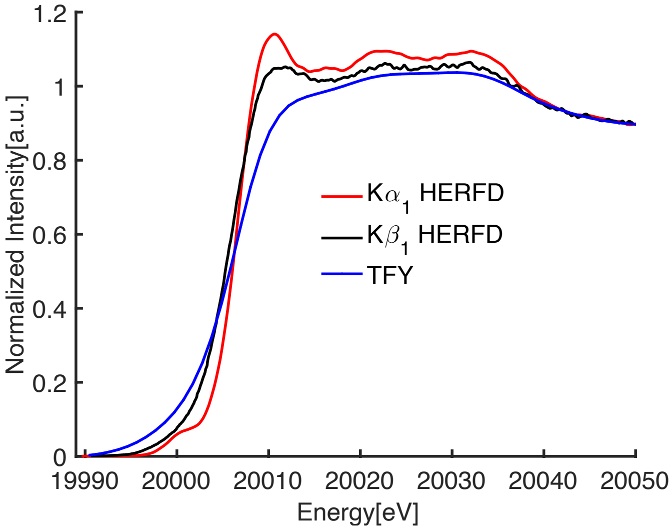

Figure S5. Mo K-edge X-ray absorption spectra of (ttcn)$MoCl_3$ measured in Total Fluorescence Yield (TFY, blue) $K\alpha_1$ detected HERFD XAS (red) and $K\beta_1$ detected HERFD XAS (black).

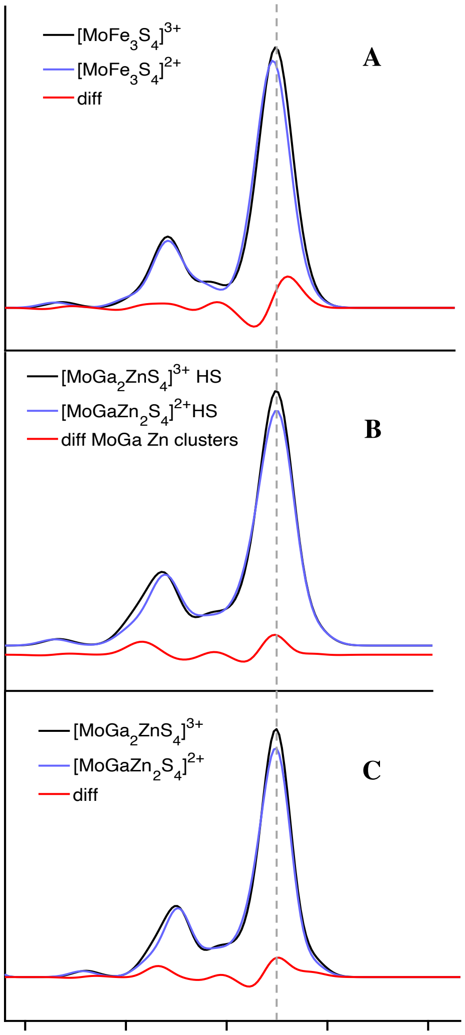

Figure S6. DFT XES calculated spectra of both MoFe cubanes (A), $[MoGa_2ZnS_4]^{3+}$ (black) and $[MoGaZn_2S_4]^{2+}$ (purple) for Mo S=3/2 (B) and Mo S=1/2 (C).

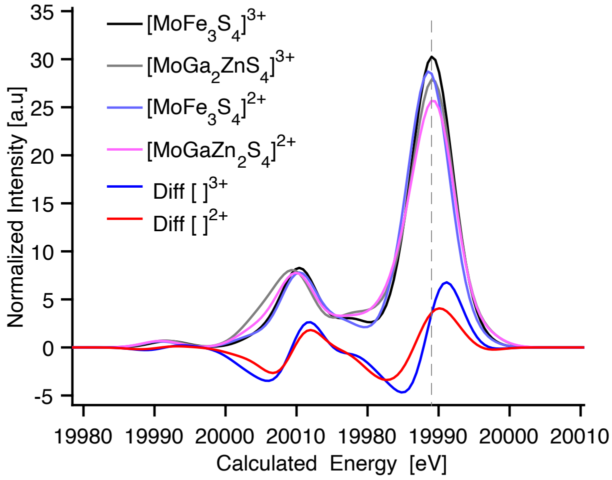

Figure S7. DFT XES calculated spectra of both MoFe cubanes, both MoGaZn cubanes and the difference spectra between cubanes of same oxidation state.

REFERENCES

1. Trofimenko, S., Transition metal polypyrazolylborates containing other ligands. *Journal of the American Chemical Society* **1969,** *91* (3), 588-595.
2. Shiu, K. B.; Curtis, M. D.; Huffman, J. C., Metal-metal multiple bonds. 15. Syntheses and structures of the 17-electron radical TpMo(CO)3 and the triply bonded dimer Tp2Mo2(CO)4(Mo.tplbond.Mo) (Tp = hydridotris(pyrazolyl)borate). *Organometallics* **1983,** *2* (7), 936-938.
3. Lincoln, S.; Soong, S. L.; Koch, S. A.; Sato, M.; Enemark, J. E., Polypyrazolylborate complexes containing the [MoSMo]2+ and [MoSeMo]2+ units. *Inorganic Chemistry* **1985,** *24* (9), 1355-1359.
4. Kowalska, J. K.; Henthorn, J. T.; Van Stappen, C.; Trncik, C.; Einsle, O.; Keavney, D.; DeBeer, S., X-ray Magnetic Circular Dichroism Spectroscopy Applied to Nitrogenase and Related Models: Experimental Evidence for a Spin-Coupled Molybdenum(III) Center. *Angew. Chem. Int. Ed.* **2019,** *58* (28), 9373-9377.
5. Fomitchev, D. V.; McLauchlan, C. C.; Holm, R. H., Heterometal Cubane-Type MFe3S4 Clusters (M = Mo, V) Trigonally Symmetrized with Hydrotris(pyrazolyl)borate(1−) and Tris(pyrazolyl)methanesulfonate(1−) Capping Ligands. *Inorganic Chemistry* **2002,** *41* (4), 958-966.
6. Solé, V. A.; Papillon, E.; Cotte, M.; Walter, P.; Susini, J., A multiplatform code for the analysis of energy-dispersive X-ray fluorescence spectra. *Spectrochimica Acta Part B: Atomic Spectroscopy* **2007,** *62* (1), 63-68.
7. Stoll, S.; Schweiger, A., EasySpin, a comprehensive software package for spectral simulation and analysis in EPR. *Journal of Magnetic Resonance* **2006,** *178* (1), 42-55.
8. Neese, F., Software update: the ORCA program system, version 4.0. *WIREs Computational Molecular Science* **2018,** *8* (1), e1327.
9. Bjornsson, R.; Neese, F.; DeBeer, S., Revisiting the Mössbauer Isomer Shifts of the FeMoco Cluster of Nitrogenase and the Cofactor Charge. *Inorganic Chemistry* **2017,** *56* (3), 1470-1477.
10. Pollock, C. J.; DeBeer, S., Valence-to-Core X-ray Emission Spectroscopy: A Sensitive Probe of the Nature of a Bound Ligand. *Journal of the American Chemical Society* **2011,** *133* (14), 5594-5601.
11. Mathe, Z.; Pantazis, D. A.; Lee, H. B.; Gnewkow, R.; Van Kuiken, B. E.; Agapie, T.; DeBeer, S., Calcium Valence-to-Core X-ray Emission Spectroscopy: A Sensitive Probe of Oxo Protonation in Structural Models of the Oxygen-Evolving Complex. *Inorganic Chemistry* **2019,** *58* (23), 16292-16301.
12. Maganas, D.; Roemelt, M.; Hävecker, M.; Trunschke, A.; Knop-Gericke, A.; Schlögl, R.; Neese, F., First principles calculations of the structure and V L-edge X-ray absorption spectra of V2O5 using local pair natural orbital coupled cluster theory and spin–orbit coupled configuration interaction approaches. *Physical Chemistry Chemical Physics* **2013,** *15* (19), 7260-7276.
13. Maganas, D.; DeBeer, S.; Neese, F., Restricted Open-Shell Configuration Interaction Cluster Calculations of the L-Edge X-ray Absorption Study of TiO2 and CaF2 Solids. *Inorganic Chemistry* **2014,** *53* (13), 6374-6385.
14. Chantzis, A.; Kowalska, J. K.; Maganas, D.; DeBeer, S.; Neese, F., Ab Initio Wave Function-Based Determination of Element Specific Shifts for the Efficient Calculation of X-ray Absorption Spectra of Main Group Elements and First Row Transition Metals. *Journal of Chemical Theory and Computation* **2018,** *14* (7), 3686-3702.
